# The transient HIV remission in the Mississippi baby: why is this good news?

**DOI:** 10.7448/IAS.17.1.19859

**Published:** 2014-11-20

**Authors:** Jintanat Ananworanich, Merlin L Robb

**Affiliations:** 1 U.S. Military HIV Research Program, Walter Reed Army Institute of Research, Silver Spring, MD, USA; 2 Henry M. Jackson Foundation for the Advancement of Military Medicine, Bethesda, MD, USA

As researchers, clinicians and community advocates gathered for the 20th International AIDS Society meeting in Melbourne in July of this year, the announcement of resumption of viremia in the Mississippi baby came as an unwelcome surprise. The case had previously stirred excitement that a cure was possible without the extreme measure of bone marrow transplantation as in the Berlin patient [1]. When this was no longer true, conversations about the end of our quest for a HIV cure ensued.

The Mississippi baby was unique in that her HIV viral load in the blood was undetectable for 27 months without antiretroviral therapy (ART) [2] – a situation that was almost unheard of in the majority of people living with HIV, for whom HIV would return with a vengeance in a matter of weeks if ART was stopped. This baby gave us hope that when ART is started so early, in her case from 30 hours of life until 18 months of age, HIV might eventually be controlled without the need for long-term ART. Such a prolonged period of viral control without ART is also termed *HIV remission* and in addition to relieving patients of the cost, inconvenience and toxicity of ART also offers hope and direction that new interventions may provide lasting viremic control or cure.

So why is this temporary HIV remission good news? First, there had been scepticism among clinicians and experts alike about whether this was true infection despite the detectable plasma viremia at five time points during her first month of life. Persaud *et al*. addressed the alternative hypotheses for the infant's infection status by showing that transfusion of maternal HIV-infected cells could not be a cause for detectable viremia without infection, and defective HIV virus or elite controller-associated HLA as causes for the viral control were similarly absent [2]. Although disappointing, the viral rebound provided unequivocal proof that the baby was indeed infected with HIV. Second and perhaps most important, it provided evidence that very early ART alone could result in a remarkably longer time to viral rebound. Third, it illustrated that the current HIV reservoir testing is not sensitive enough to measure very low levels of HIV persistence. The Mississippi baby had no detectable replication-competent virus in resting CD4+ T cells, but yet, HIV came back when suppressive ART was removed. This is in concordance with another transient HIV remission report of two adults, also called the Boston patients, who had bone marrow transplantation for cancer from CCR5+ donors. After transplantation, while on ART, they had no detectable HIV viremia or cell associated HIV DNA but the viral load rebounded in 3 months in one patient and 7 months in the other after ART was stopped [3].

Recently, Canadian researchers presented a case that was treated within the first 24 hours of life and was on ART for 3 years before treatment was interrupted. Although the baby had neither detectable replication-competent virus or proviral DNA, the plasma viral load quickly returned and rose to over 100,000 copies/ml following ART interruption [4]. Another case, the Milan baby initiated ART at 12 hours of life until age 3 years, and subsequently, had a viral rebound within 2 weeks of interruption [5]. What was different in these cases compared to the Mississippi baby (Table 1)? The clue may be in the amount of time it takes to achieve viral suppression. The Mississippi baby's viral load was below 50 copies/ml within the first month of life while it took longer in these children. Ability to suppress viral load in a short time correlates with lower reservoir size [6]. The Milan baby also had a high viral load initially (152,560 copies/ml) raising a possibility for more HIV seeding of blood and tissue reservoirs. The Canadian baby showed evidence of on-going viral replication with detectable cell-associated HIV RNA. Markers for such replication were also evident in the Milan baby who had detectable HIV-specific CD4+ and CD8+ T cells, higher frequencies of activated T cells and altered proportions of naïve and memory CD4+ T cell subsets prior to treatment interruption. There are four other Canadian babies and a baby from Long Beach, California, who initiated ART within hours after birth and currently have no detectable proviral DNA [4,7,8]. There are also four adolescents with viral suppression for the past 15–17 years since initiating ART in infancy with a similar profile [9]. It is unclear whether these children will achieve HIV remission as they have not yet interrupted ART.

**Table 1:** Early treated children with different time to HIV viral rebound

Parameters	Mississippi [2]	Canadian [4]	Milan [5]
Time to viral rebound	27 months	<1 month	<1 month
ART onset	30 hours	<24 hours	12 hours
Pre-ART HIV RNA. copies/ml	19,812	808	152,560
Time to HIV RNA <50 copies/ml on ART	1 month	6 months	3 months
Time on ART before interruption	18 months	3 years	3 years
Cell-associated HIV DNA	Undetected^a^	Undetected	Undetected
Replication-competent virus	Negative viral outgrowth assay^a^	Negative viral outgrowth assay	Negative viral culture
HIV antibody	Non-reactive^a^	Non-reactive	Non-reactive
HIV-specific T cells	Undetected^a^	Not reported	Detected
Others	Normal frequencies of activated T cells^a^	Detected cell-associated HIV RNA	High frequencies of activated T cells

a The reservoir and immunity testing in the Mississippi baby were performed after ART was interrupted. The testing on the Canadian and Milan babies was performed during ART. ART: antiretroviral therapy.

HIV persistence occurs when infected cells revert to a resting state instead of continuing its usual path from activation to death. These small numbers of quiescent HIV infected cells, mostly central memory CD4+ T cells, persist indefinitely and are not eliminated by the HIV medications or by the immune system. The cellular reservoir can be quantified by its HIV DNA content, which is mostly defective with only 0.1% estimated to be replication competent after a single round of activation in the viral outgrowth assay. Currently not measured by standard assays is the larger portion of HIV-DNA-positive cells that could produce virions with additional rounds of activation [10].

All these cases have taught us that the amount of the latent virus, the markers for on-going viral replication and the HIV-specific immunity may be important in achieving HIV remission. The latent virus as measured by HIV DNA was too low to be detected by current methods in all cases but the virologic and immunologic markers for on-going viral replication in the Canadian and the Milan babies cannot be ignored. The absent HIV-specific immunity in the Boston patients and the Mississippi baby possibly resulted in their inability to eliminate the few infected cells, and with time, active viral replication occurred. The detectable HIV-specific T cells in the Milan baby does not equate to an effective immunologic responses against HIV but merely a marker for active HIV presence. It is clear that we have much to learn. These cases present us with an opportunity to improve outcome by employing strategies to further reduce the HIV reservoir and boost immunity against HIV. Starting ART earlier and treating for longer with drugs that have better penetration while giving therapeutic HIV vaccination, broadly neutralizing antibody or anti-programmed cell death-1 antibody, are amongst the potential strategies [11].

The task of eliminating every single infected cell to achieve HIV eradication is daunting and not possible with current therapies but the short-term goal of HIV remission may be possible if the reservoir can be sufficiently reduced [12]. Early ART is to date the most effective strategy to reduce the latent reservoir. Additionally, at birth, newborns lack memory CD4+ T cells [13], infection of which represents a major barrier to cure. These provide a strong rationale to initiate ART as soon as possible during this window of opportunity in infants. This is a goal that will not only prevent death and improve health but may also move our children one step closer to a HIV cure.
